# The Relationship Between Housing, Life-Course Transitions, and Old Age Social Exclusion: A Cross-Country Analysis

**DOI:** 10.1093/geroni/igaa057.2002

**Published:** 2020-12-16

**Authors:** Anna Wanka, Urbaniak Anna, Kieran Walsh, Frank Oswald

**Affiliations:** 1 Goethe University Frankfurt am Main, Frankfurt am Main, Hessen, Germany; 2 University of Vienna, Vienna, Wien, Austria; 3 Irish Centre for Social Gerontology, National University of Ireland Galway, Galway, Galway, Ireland; 4 Interdisciplinary Ageing Research, Frankfurt, Hessen, Germany

## Abstract

The international literature presents growing evidence of the impact of life transitions in older age on experiences of social exclusion, and place in general and as well as housing in particular potentially play a mediating role in this interrelation. However, the specific mechanisms through which the older adult place relationship mediates exclusionary outcomes of life-course transitions remains poorly understood in the study of ageing. This contribution investigates how older adults’ relationship to their home is interlinked with life-course transitions and old-age social exclusion. To do so, we present case studies from three different countries (Germany, Ireland and Poland), focusing on the individual experiences of retirement and bereavement, and analyze them by drawing on the concepts of the person-environment exchange processes of agency and belonging. Finally, we draw conclusions about how spatial agency and belonging can protect and empower older people at critical junctures in their lives.

